# COVID-19 pandemic and ophthalmological emergencies: a case-control analysis of the impact of lockdowns in a University Hospital in Lombardy region, Italy

**DOI:** 10.1080/07853890.2023.2248882

**Published:** 2023-08-24

**Authors:** Simone Donati, Edoardo Appolloni, Sebastiano Ceriani, Elias Premi, Laura Premoli, Cristian Metrangolo, Arianna Ravasio, Claudio Azzolini, Paolo Radice

**Affiliations:** a Department of Medicine and Surgery, University of Insubria, Varese-Como, Italy; b U.O.C. Oculistica, Ospedale di Circolo e Fondazione Macchi, ASST Sette Laghi, Varese, Italy; c Department of Neurological and Vision Sciences, Eye Clinic, University of Brescia, Brescia, Italy; d Department of Biotechnologies and Life Sciences, University of Insubria, Varese-Como, Italy

**Keywords:** COVID-19 lockdown, ophthalmology emergencies, ocular trauma

## Abstract

**Purpose:**

To evaluate the incidence of ocular pathologies seen at the ophthalmological emergency department (OED) during the national lockdown in 2020 due to the COVID-19 pandemic and compare it to the corresponding period in 2019.

**Methods:**

Electronic records of patients who presented at the OED of our University Hospital in Varese, Italy during the COVID-19 lockdown were compared with that from the corresponding period in 2019. Records from the spring (2020A) and winter (2020B) lockdowns were compared with each other and with the same periods in 2019 (2019A and 2019B). Statistical analyses were performed by unpaired Student’s *t*-tests, Poisson’s regression and Chi-square test.

**Results:**

The number of consultations at the OED significantly decreased during the COVID-19 lockdown (*p* value <.0001). The largest decreases were observed in the youngest (age <15 years: –77.3%) and oldest (age >61 years, –68.5%) age groups. The proportion of men who consulted increased significantly from 61.76% in 2019A to 67.63% in 2020A, and from 54.56% in 2019B to 62.79% in 2020B. A significant reduction in deferrable consultations was also reported (from 943 in 2019 to 335 in 2020; *p* value <.0001). A statistically significant decrease in the number of consultations involving ocular trauma was also reported despite an increase in its proportion among all consultations for ocular pathologies in 2020.

**Conclusions:**

Our evaluation showed a significant reduction in the number of OED consultations in all deferrable pathologies. Although the incidence of conditions that affect visual function was lower, these were more frequent in the lockdown period. The significant reduction in the number of deferrable consultations highlights the misuse of the OED.

## Introduction

The COVID-19 pandemic forced changes in lifestyle and habits, leading to changes in social behaviour [1]. The imposed lockdowns also influenced the management and healthcare activities of hospitals and clinics [2,3]. One of the affected healthcare services was access to the emergency room, wherein changes in the workplace structure impacted its services [4]. Additionally, restrictions in traveling, and movements of people highlighted several aspects in the mismanagement and misuse of healthcare services [5].

The first case of COVID-19 in Italy was documented on 21 February 2020 in Codogno, Lombardy when an Italian patient was confirmed as having SARS-Cov-2 [6]. Subsequently, Italy, including Lombardy, imposed two lockdown periods to limit the movement of people in an attempt to minimize viral transmission [7]. The first lockdown was imposed in spring, while the second was imposed in winter. People were forced to stay at home, all ‘non-essential’ businesses closed, and many companies shut down; remote work was introduced. All restrictions impacted the daily lives of the population as well as the clinical practice in hospitals [8].

During the first lockdown, all scheduled visits, and ordinary surgical activities were postponed. During the second lockdown, some scheduled clinical activities were allowed but with stringent measures to prevent the transmission of COVID-19 to healthcare personnel and patients. Emergency surgery was the only medical treatment performed during both periods [9].

The aim of our study was to determine the impact of the pandemic and lockdowns on the consultations for emergency services at our University Hospital. Moreover, we attempted to identify the effects that the pandemic and lockdowns had on everyday life. Finally, we studied how ophthalmological emergencies reflected the changes in lifestyle brought about by the pandemic.

## Materials and methods

This retrospective, single-centre, cross-sectional study was conducted to analyse consultations to the ophthalmological emergency services of ASST Sette Laghi ‘Ospedale di Circolo e Fondazione Macchi’ University Hospital in Varese during the COVID-19 lockdowns in 2020–2021. We analysed records of patients from 7 March 2020 to 4 May 2020 (indicated as 2020A), which corresponded to the spring lockdown, and compared them with those in the same period in 2019 (indicated as 2019A).

Similarly, we analysed records from 6 November 2020 to 6 January 2021 (indicated as 2020B), which corresponded to the winter lockdown, and compared them with those in the same period in 2019 (2019B). We collected demographic and clinical data of patients from the electronic database of our hospital. SC, EA and EP collected data from the central hospital database. SD and CM controlled the data, which were collected consistently by verifying research engines and keys. All data were classified and organized in a statistical datasheet for analysis. Each pathology and clinical condition were coded using the International Classification of Diseases, 9th Revision (ICD-9-CM).

Patients were divided into four groups based on age: <15 years old, 16–30 years old, 31–60 years old and >60 years old. Clinical diagnosis was classified into five groups: anterior segment (AS), posterior segment (PS), neuro-ophthalmology (NO), orbits/annexa (OA) and others (Ot) (if unspecified visual disturbances were defined as access diagnosis). We also identified the nature of traumatic conditions, including trauma due to foreign body and contusive trauma. Finally, we categorized the severity of each condition as deferrable or non-deferrable consultations.

The study was approved by the ethics committee of ATS Insubria. Informed consent was obtained from each patient for data collection, storage and analysis upon acceptance at the triage service of the emergency department.

This study was submitted to CLINICALTRIALS.gov and approved with the following code: NCT05065788.

### Statistical analysis

To compare trends in the absolute number of emergency department consultations, we considered Poisson’s regression models as appropriate for the count nature of dependent variables. Under this log-linear approach, time trends can be approximated by 100 × (exp(*β*) – 1), where *β* is the period coefficient in the model. We also reported the Wald Chi-square test *p* values to test the homogeneity of the number of consultations by period.

The association between the prevalence of emergency department consultations during the study periods and patient characteristics (age and sex) or by type and presence of trauma was investigated using the Chi-square test. All statistical analyses were performed using SAS 9.4 (SAS Company, Cary, NC).

## Results

**Figure 1.**
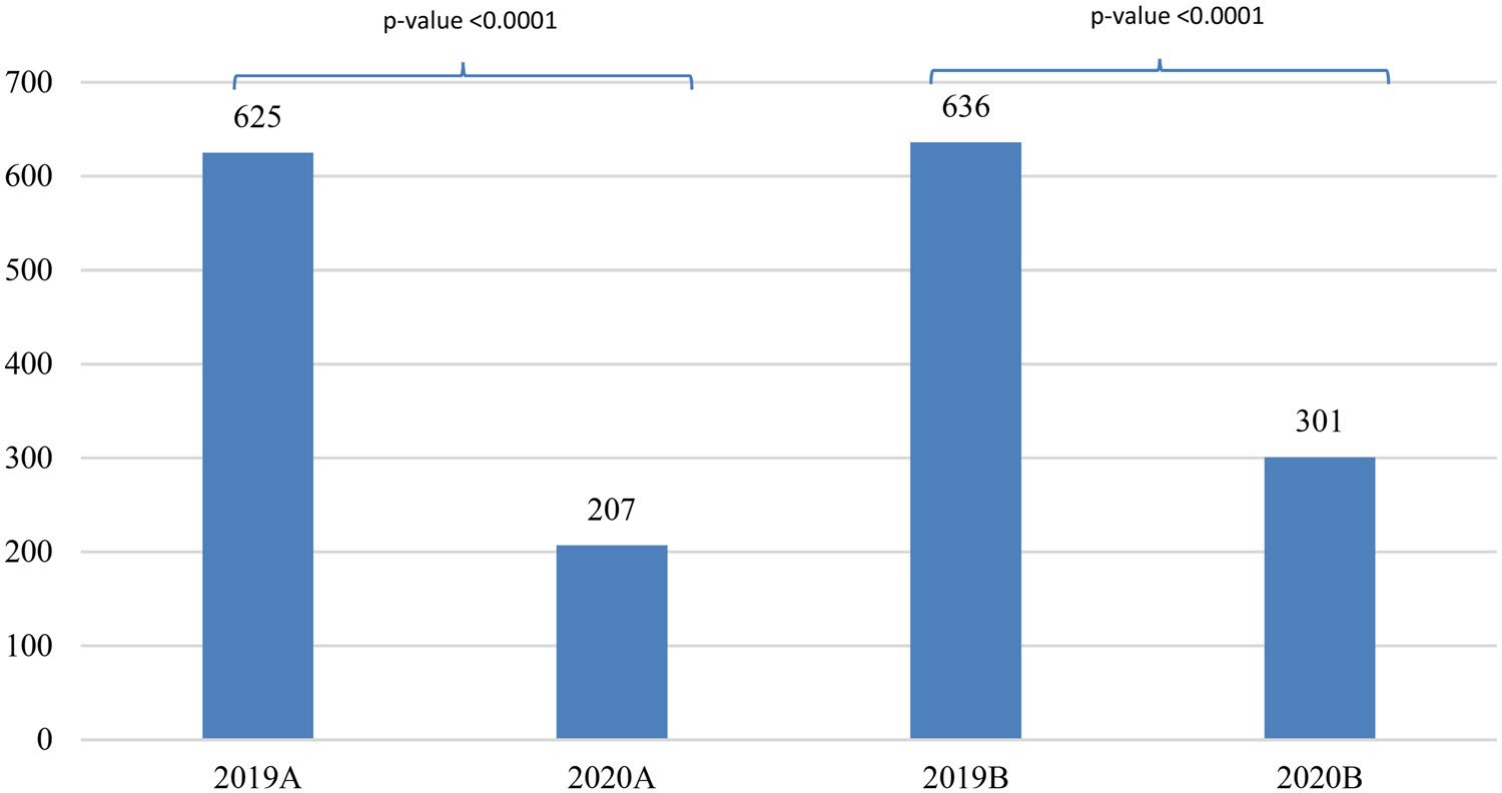
Total number of consultations to the ophthalmological emergency department according to the studied period.

There was a significant decrease in the total number of consultations during the COVID-19 lockdown periods when compared to the same periods before the COVID-19 pandemic. In 2020, the number of consultations was 508 vs. 1261 in 2019, which is a decrease of approximately 60% (*p* value <.0001); this trend was maintained if we compared the corresponding periods (Figure 1).

Moreover, in 2020, the variation between the two lockdown periods was also significant. There were 207 consultations in 2020A vs. 301 in 2020B (41.54%; *p* value <.0001).

**Figure 2.**
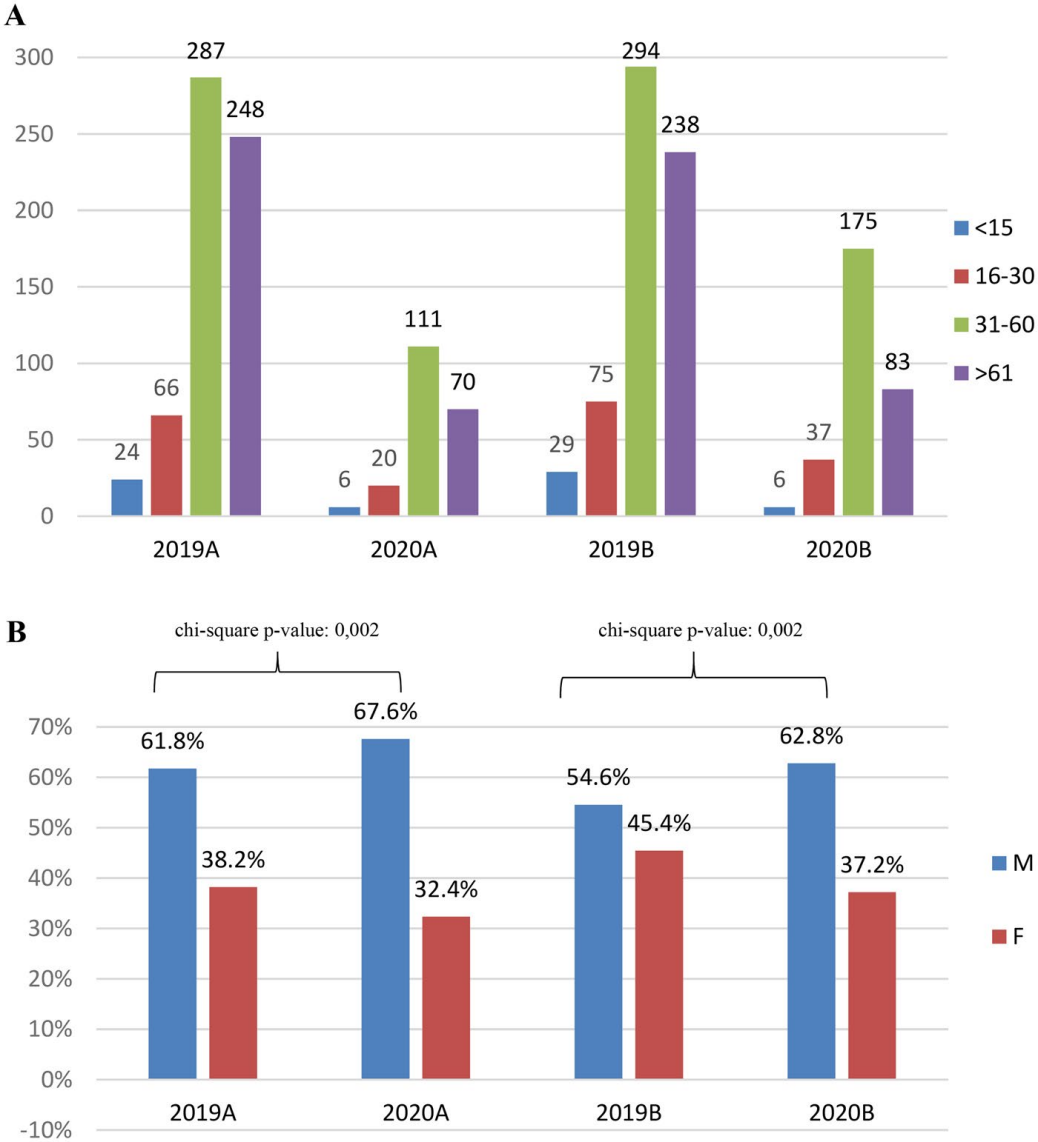
(A) Number of consultations according to age during the study periods. (B) Number of consultations according to sex during the study periods (M: males; F: females).

Figure 2 shows the patient characteristics according to sex and age. There was no significant difference in the age distribution of patients during the study periods (*p* value = .15). In 2019A, the mean age ± standard deviation of patients was 54.0 ± 20.2 years vs. 52.3 ± 18.4 in 2020A; in 2019B, it was 52.7 ± 20.8 vs. 50.8 ± 18.0 in 2020B.

There were interesting findings regarding the number of consultations according to age group (*p* value = .0004). The two age groups that showed the largest decrease in the number of consultations were patients aged <15 years (53 in 2019 vs. 12 in 2020, –77.3%) and those aged >61 years (486 in 2019 vs. 153 in 2020, –68.5%). Meanwhile, the decrease in the number of consultations in the other age groups was not as remarkable (16–30 years old: 141 in 2019 vs. 57 in 2020, –59.6%; 31–60 years old: 581 in 2019 vs. 286 in 2020, –50.8%).

Meanwhile, the proportion of consultations from patients aged 31–60 years significantly increase from 45.92% in 2019A to 53.62% in 2020A, and from 53.62% in 2019B to 58.14% in 2020B. In contrast, that from patients aged >61 years significantly decreased from 39.68% in 2019A to 33.82% in 2020A, and from 37.42% in 2019B to 27.57% in 2020B (Figure 2(A)).

In terms of the sex, the proportion of male patients who consulted in the emergency department increased in both periods in 2020 (Figure 2(B)).

**Figure 3.**
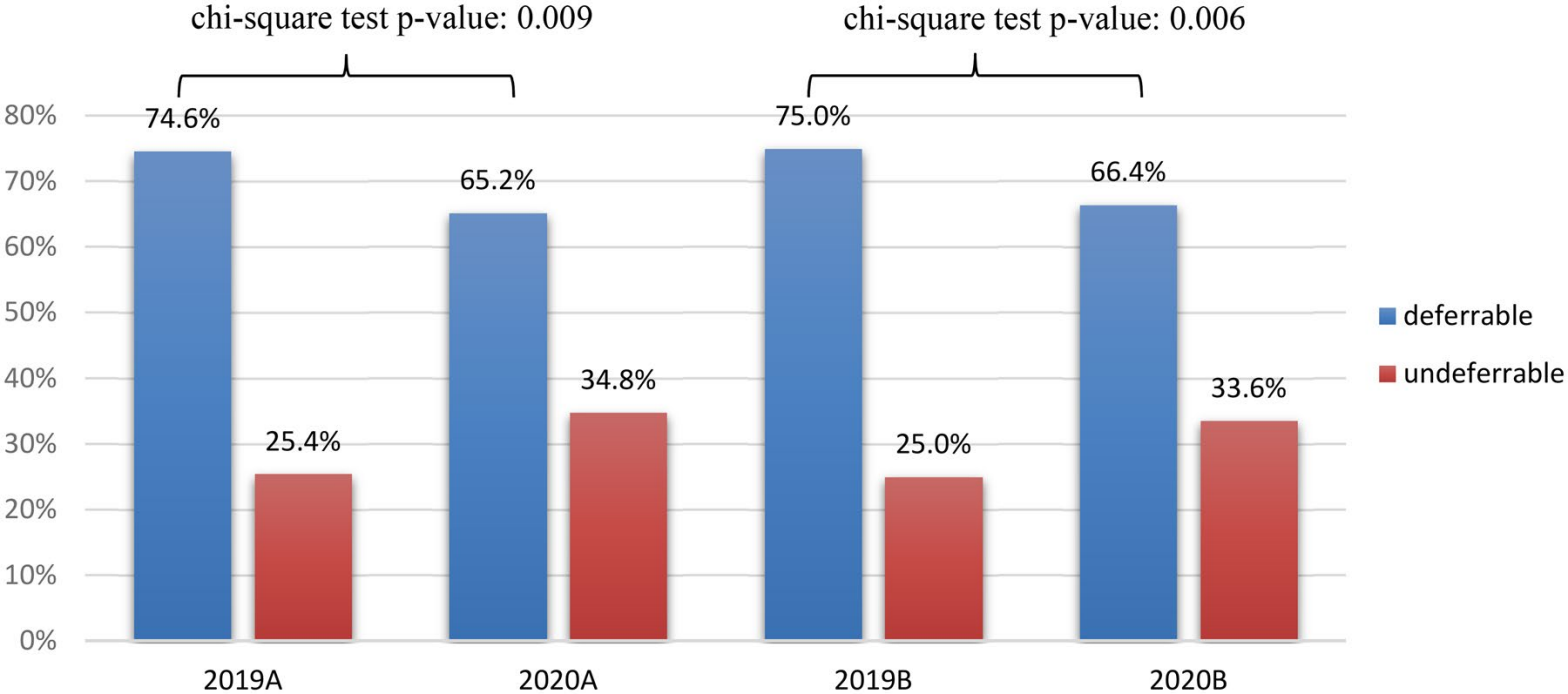
Percentage of non-deferrable and deferrable consultations in 2019 and 2020.

Notably, there was a reduction in the number of both deferrable (943 in 2019 vs. 335 in 2020; *p* value <.0001) and non-deferrable (318 in 2019 vs. 173 in 2019; *p* value <.0001) consultations. Additionally, analysis showed that the proportion of emergency consultations increased from 25.22% in 2019 to 34.06% in 2020 (*p* value = .0002). Figure 3 shows the same values for the selected periods.

**Figure 4.**
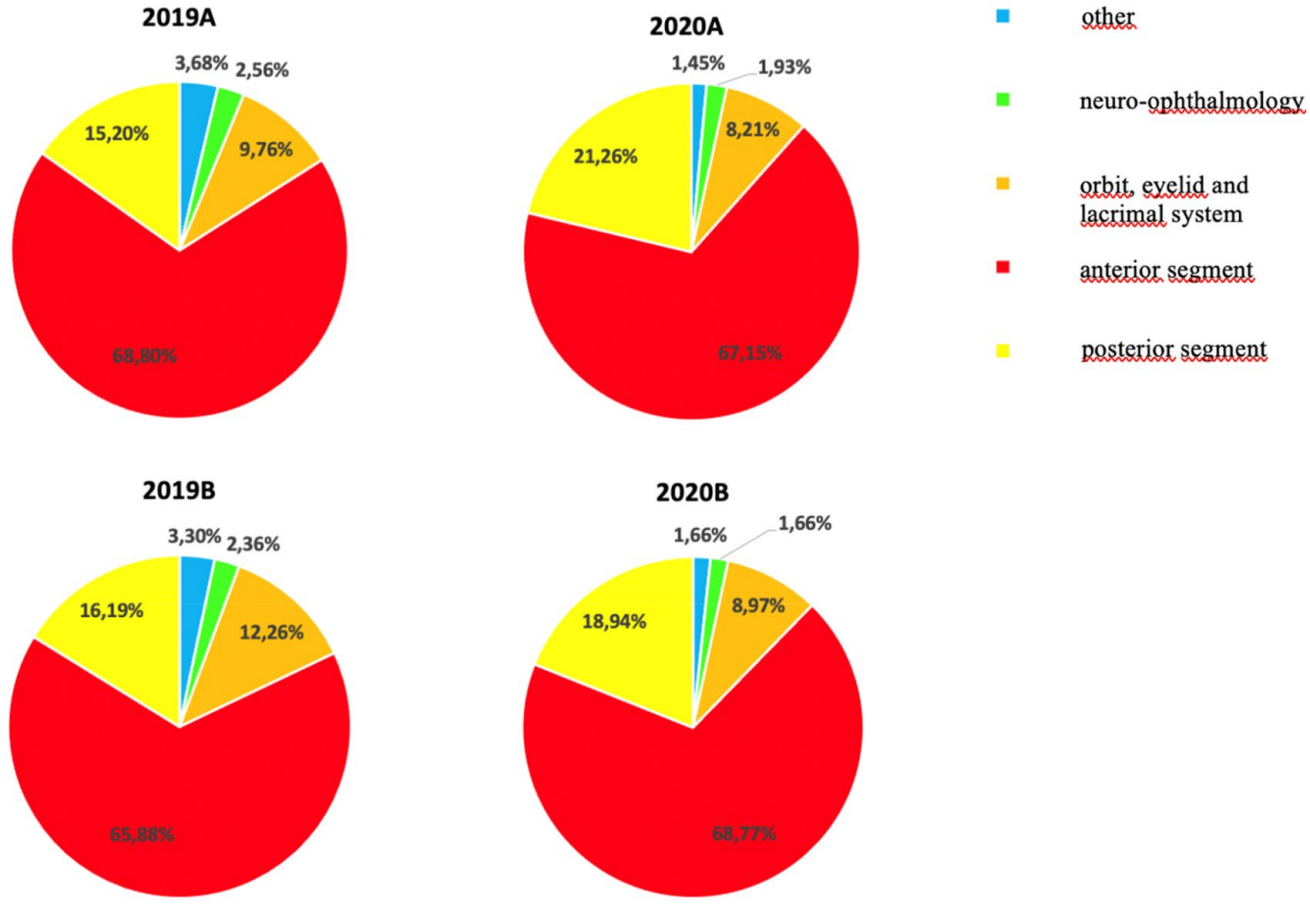
Affected ocular regions upon consultation during the selected periods. The anterior segment was the most involved in all consultations in 2019 (67.33%) and 2020 (68.11%) (*p* = .75). The proportion of consultations involving the posterior segment significantly increased from 15.20% in 2019A to 21.26% in 2020A, and from 16.19% in 2019B to 18.94% in 2020B (*p* value = .04, *p* value = .30, respectively). The proportion of consultations involving the orbit and annexa did not significantly change between 2019 and 2020 (2019A, 9.76% vs. 2020A, 8.21%; 2019B, 12.26% vs. 2020B, 8.97%). The proportion of consultations involving for neuro-ophthalmology decreased from 2.46% in 2019 to 1.77% in 2020 (2019A, 2.56% vs. 2020A, 1.935; 2019B, 2.36% vs. 2020B, 1.66%). The proportion of consultations involving for other ocular regions also decreased from 3.68% in 2019A to 1.45% in 2020A, and from 3.30% in 2019B to 1.66% in 2020B.

We then evaluated the affected regions of the eye during consultations as OA, AS, PS, NO and Ot (Figure 4**)**. Our data show that most consultations involved the AS in both 2019 and 2020 (2019, 67.33%; 2020, 68.11%) (*p* value = .75). The PS was involved in 19.88% and 15.70% of all consultations in 2019 and 2020, respectively (*p* value = .03). Consultations involving the OA decreased from 11.02% in 2019 to 8.66% in 2020. Meanwhile, the number of consultations involving the NO and Ot were not significantly different between 2019 and 2020 (NO, 2.46% in 2019 vs. 1.77% in 2020; Ot, 3.49% in 2019 vs. 1.57% in 2020).

**Figure 5.**
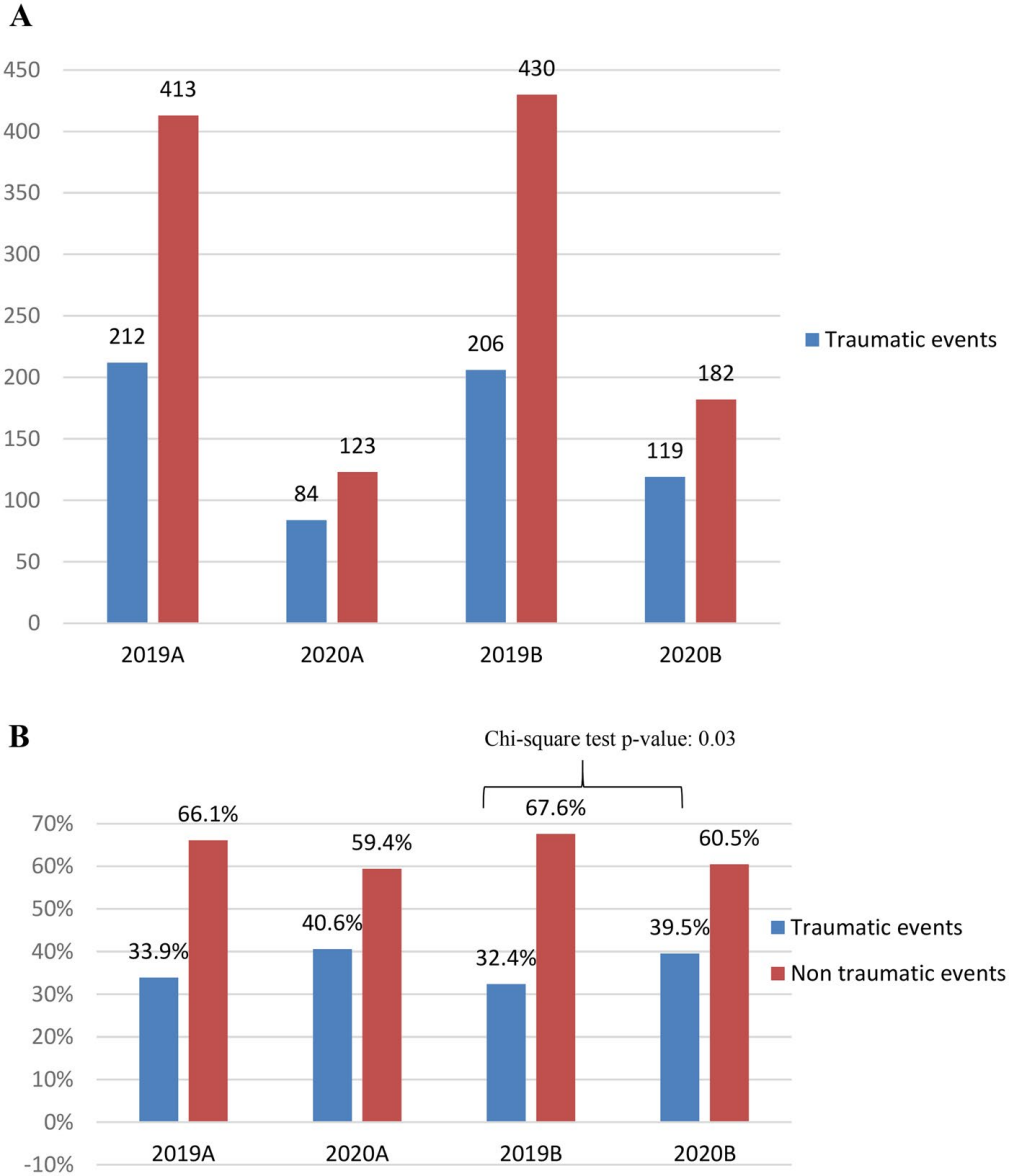
Incidence of traumatic and non-traumatic pathologies. (A) Number of events (2019 vs. 2020: *p* value <.0001); (B) relative percentages of events (traumatic events: 2019B vs. 2020B, *p* = .03).

**Figure 6.**
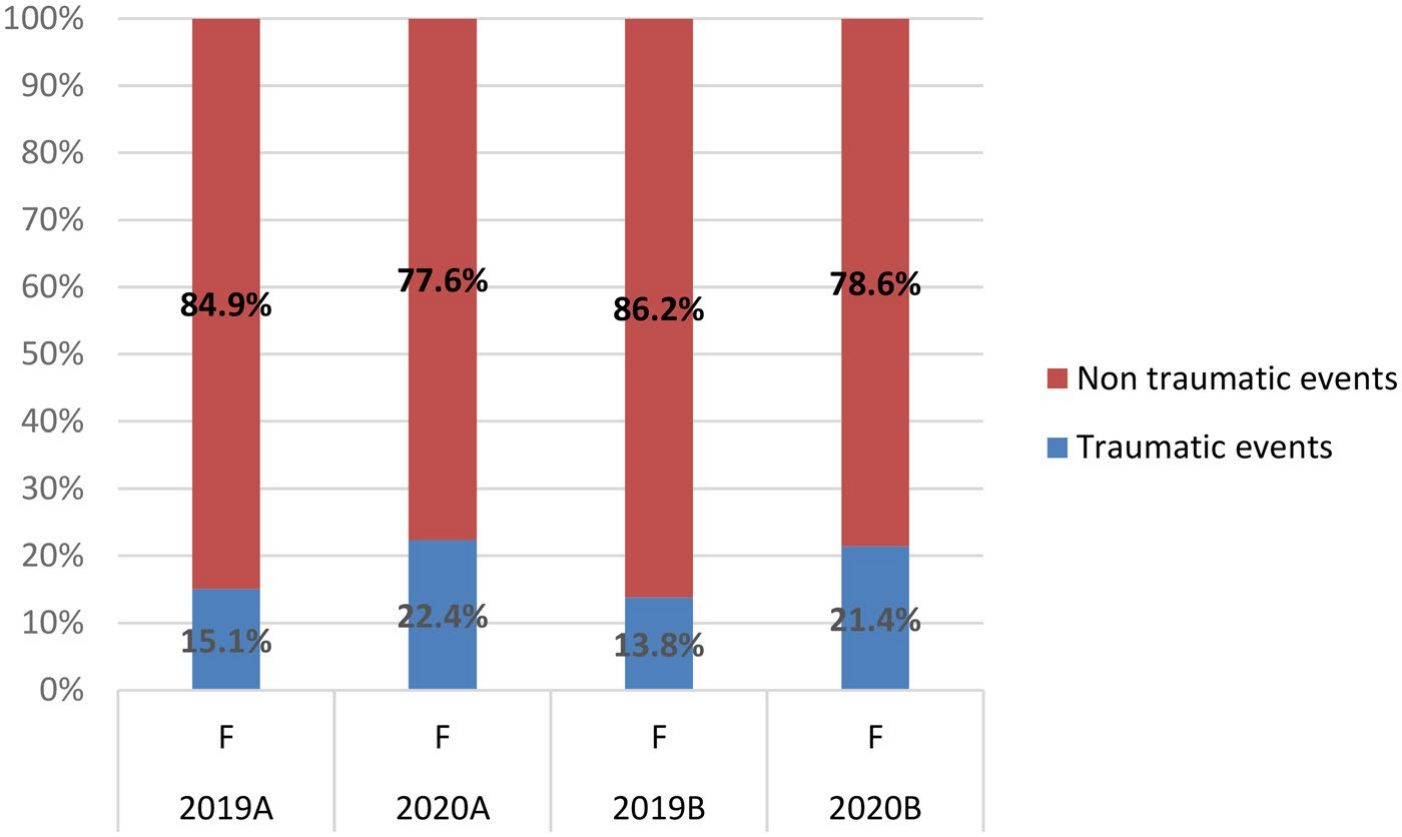
Incidence of traumatic and non-traumatic events in female patients.

Regarding ocular trauma, there was a significant decrease in the number of emergency department consultations due to ocular trauma (418 in 2019 vs. 203 in 2020; *p* value <.0001) (Figure 5(A)). However, the proportion of consultations that involved ocular trauma increased in 2020. In 2019A, 33.92% of all consultations involved ocular trauma vs. 40.58% in 2020A (*p* value = .08); similarly, in 2019B, 32.39% of all consultations involved ocular trauma vs. 39.53% in 2020B (*p* value = .03) (Figure 5(B)). Additionally, analysis revealed that the proportion of male and female patients who consulted due to ocular trauma both increased in 2020 (Figure 6).

The number of consultations in 2019A was similar to that in 2019B (*p* value = .77). Meanwhile, the number of consultations from the first lockdown to the second lockdown significantly increased by 41% in 2020 (84 vs. 119; *p* value = .015).

## Discussion

The COVID-19 pandemic significantly modified people’s habits as well as the implementation of healthcare services [10,11]. This study revealed that compared with that in 2019, the number of ophthalmological consultations in the emergency department of our hospital in 2020 decreased by 60%. Posarelli et al. highlighted the downward trend in the number of consultations from 2019 to 2020 in their analysis of ophthalmological consultations at the emergency department of Azienda Ospedaliero-Universitaria Pisana [12].

According to previous reports [13,14], one of the primary reasons for the decrease in the number of consultations, particularly in hospitals, was due to the fear of being infected with COVID-19. Hospital and healthcare institutions were seen as potential areas of transmission of the disease. Hence, patients lost confidence in the safety of healthcare institutions and only consulted if urgently needed.

Another reason that influenced the number of consultations is the periodic lockdowns and their level of implementation. Data showed that the number of patients who consulted to the emergency department in 2020B was approximately 45% higher than that in 2020A, when the lockdown was more stringent and stricter. The increase in the number of consultations was not related to the season as this difference was not observed in 2019; from 2019A and 2019B, the number of consultations only increased by 1.76%. In 2020A, movement of people was limited due to the stringent measures implemented in Red Areas due to a higher number of cases and greater risk of transmission [15,16]. In 2020B, the reduction in the number of cases allowed for the modification of the limitations. The restrictions resulted in people being wary of their safety, leading to the avoidance of healthcare institutions except in emergency cases.

To evaluate the severity of emergency consultations, we classified patients according to whether consultation was deferrable and non-deferrable. The number of consultations for both types of consultations decreased during the pandemic, probably due to the same reasons that led to the reduction in the total number of consultations. However, the number of non-deferrable conditions increased in 2020. Consequently, the number, and proportion of consultations due to deferrable conditions decreased. These trends suggest that due to fear of contracting COVID-19, patients only consulted to the emergency department when their ophthalmological conditions were acute and severe enough that it affected their daily activities [17–20].

Regarding the affected ocular region, the number of consultations between 2019 and 2020 did not significantly differ except for those involving the PS, which increased from 15.70% in 2019 to 19.88% in 2020. We think that because pathologies involving the PS generally cause signs or symptoms involving visual acuity, these prompt patients to seek emergency consultation despite the fear of contracting COVID-19. However, the stringent lockdown measures influenced the number of consultations as the difference in the number of consultations between 2019A and 2020A was greater than that between 2019B and 2020B.

The number of consultations due to ocular trauma decreased from 2019 to 2020. This trend was also observed when the corresponding two periods in 2020 and 2019 were compared. However, if we only consider the proportion of consultations due to ocular trauma, our analysis revealed a significant increase from 2019B to 2020B. The increase could be explained by analysing the causes of trauma before and after the COVID-19 pandemic [21,22]. The restriction in commercial and productive activities in both 2020A and 2020B may have led to a limitation in potentially dangerous activities that may cause ocular trauma. However, this trend was not reported in our analysis, as described.

Pellegrini et al. [21] analysed the differences between the causes of trauma before and during the COVID-19 pandemic. They found that the incidence of ocular injuries caused by falls and sports activities decreased, but that caused by home and gardening activities increased. This suggests that due to the restrictions imposed, the incidence of work, and daily activity-related ocular trauma decreased, but due to people staying at home, the incidence of ocular trauma related to home activities, such as gardening, increased.

Our study also revealed that there was no significant difference in the incidence of ocular trauma-related consultations between 2019A and 2020A. However, there was a significant difference in the incidence of ocular trauma-related consultations between 2019B and 2020B. In 2020B, as the lockdown was less severe than in 2020A, some activities resumed, and home activity-related trauma was seen with job-related trauma in the emergency department, resulting in a significant increase in the proportion of patients with ocular trauma from 2019B to 2020B (*p* < .05).

Regarding age, the number of consultations decreased in all age groups from 2019 to 2020. However, the decrease was more pronounced in some age groups. Patients aged <15 years had the greatest decrease in the number of consultations (–77.3%), which could be related to the interruption of school activities during the lockdown. The interruption may have even protected children from ocular pathologies such as ocular surface infective pathologies and trauma. Consultations of patients aged 16–30 years decreased by 59.6%, which may be attributed to the interruption of sports and outdoor activities. Meanwhile, consultations of patients aged >61 years decreased by 68.5%. Notably, this age group had the highest risk for complications caused by COVID-19. The fear of being infected forced these patients to stay indoors and lessen socialization and activities, resulting in these patients only consulting for chronic or serious ocular pathologies [23–26]. The decrease in the number of consultations was the smallest among patients aged 31–60 years (–50.8%). Most of these patients belong to the workforce; hence, they may have continued working, with some of them still being exposed to potential causes of ocular pathologies and trauma. This may also be the reason why consultations of patients in this age group due to trauma did not significantly decrease.

This study has some limitations. First, this was a retrospective study; its design may have inherent limitations. Moreover, as our analysis evaluated different periods and cohorts of patients, our results may not be generalized due to different countries having different restrictions during the pandemic. Additionally, the definition of deferrable and non-deferrable pathologies was subjective and depended solely on the clinician as there is no consensus on what pathologies are deferrable or non-deferrable.

In conclusion, the COVID-19 pandemic radically changed how emergency departments operate. Lockdowns highlighted some aspects regarding emergency ophthalmological consultations and its misuse. The significant reduction in deferrable consultations indicates the behaviour of patients that seek emergency consultations despite minor ocular concerns. Meanwhile, many patients did not consult despite having an ocular pathology due to fear of contracting COVID-19 [27]. Another notable finding is the decrease in the number of non-deferrable consultations, especially for cardiac pathologies [28]. Although people were less exposed to environmental risk factors, they opted to stay home instead of visiting the emergency department due to fear of contracting COVID-19 despite having a non-deferrable pathology. The results of our study may be confirmed after a few years when hospital operations return to normal [29–31]. To encourage patients to consult for any pathology and increase their confidence in healthcare institutions, the implementation of safety measures should be continued and encouraged.

## Data Availability

Data available on request from the authors.
